# Correction: An empirical analysis of the demand for family planning satisfied by modern methods among married or in-union women in Nigeria: Application of multilevel binomial logistic modelling technique

**DOI:** 10.1371/journal.pone.0307782

**Published:** 2024-07-22

**Authors:** The PLOS ONE Staff

In Fig 1, the information “Sources” is missing. Please see the correct Fig 1 here.

**Fig 1:**
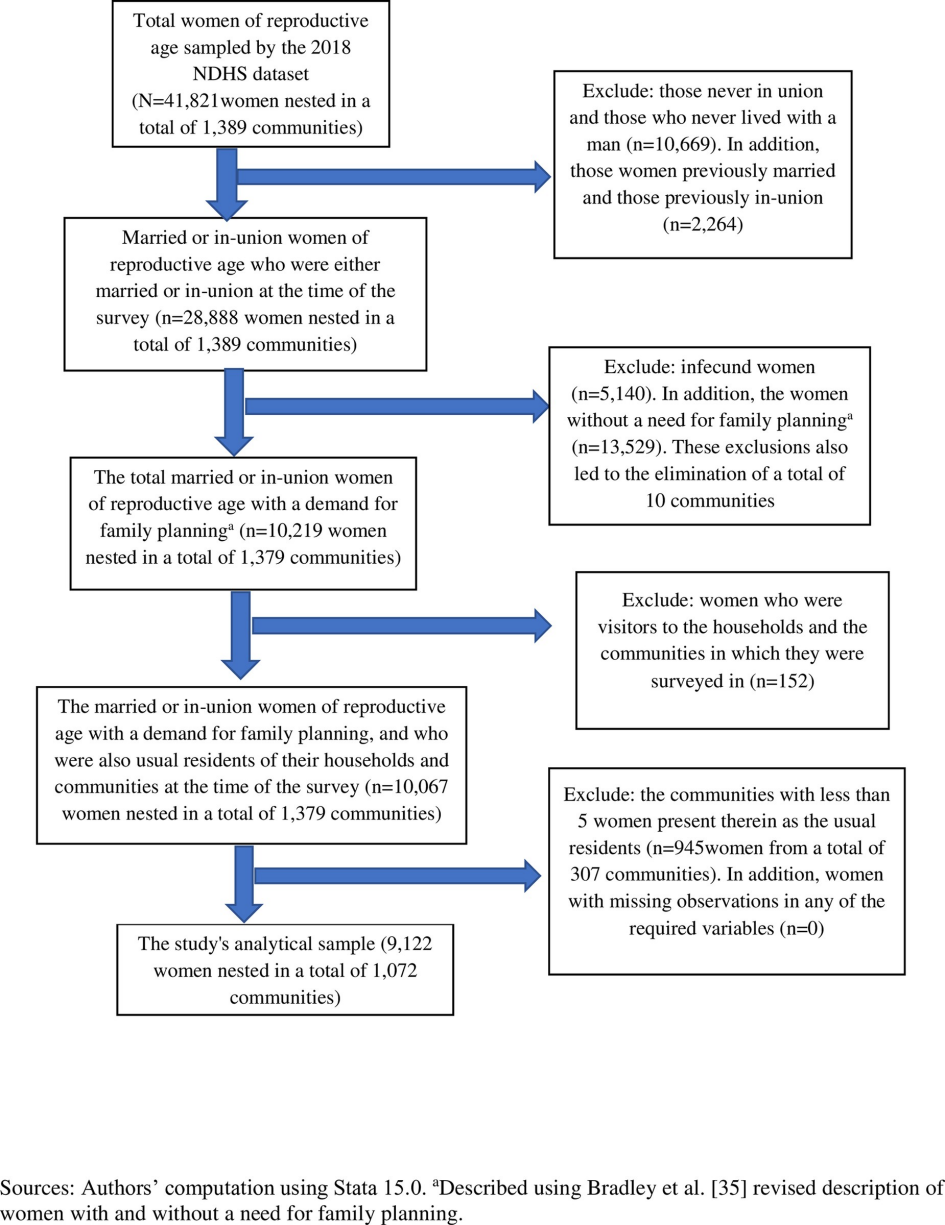
Flow chart for obtaining this study’s analytical sample.

The Table 3 is formatted incorrectly. Please see the correct Table 3 here.

**Table 3:** Results for model Ds^a^.

Independent variables	aORs (95%CI)	SERs	p value
***Individual-level (level 1) variables***			
**Age group of women:**			
15 to 19(ref.)	1.00		
20 to 24	1.359(0.843 to 2.189)	0.330	0.207
25 to 29	1.768(1.110 to 2.817)	0.420	0.016**
30 to 34	1.624(1.005 to 2.624)	0.397	0.047**
35 to 39	1.700(1.002 to 2.887)	0.459	0.049**
40 to 44	1.677(1.009 to 2.788)	0.434	0.046**
45 to 49	1.375 (0.756 to 2.499)	0.419	0.296
**Ethnicity:**			
Hausa/Fulani/Kanuri (ref.)	1.00		
Yoruba	1.839(1.220 to 2.771)	0.384	0.004***
Igbo	1.553(0.969 to 2.490)	0.373	0.067*
others	1.621(1.181 to 2.224)	0.261	0.003***
** Total number of children alive:**			
0 children alive (ref.)	1.00		
1 to 4 children alive	2.614(1.266 to 5.398)	0.966	0.009***
more than 4 children alive	2.661(1.233 to 5.742)	1.043	0.013**
** Ideal number of children:**			
0 child (ref.)	1.00		
1 to 4 children	2.163(1.302 to 3.594)	0.560	0.003***
more than 4 children	1.873(1.137 to 3.085)	0.476	0.014**
**Highest educational level attained by the husbands/partners:**			
no formal education (ref.)	1.00		
primary education	1.828(1.389 to 2.405)	0.256	<0.001***
secondary education	2.048(1.575 to 2.662)	0.274	<0.001***
higher education	2.326(1.659 to 3.260)	0.400	<0.001***
**Age of women at first marriage/cohabitation:**			
<18 years (ref.)	1.00		
18 to 30 years	0.944(0.797 to 1.118)	0.081	0.505
31 to 49years	0.476(0.304 to 0.745)	0.109	0.001***
**Money for medical care:**			
yes, it is a big problem (ref.)	1.00		
vno, it is not a big problem	1.128(0.966 to 1.317)	0.089	0.128
** Exposure to family planning messages through various media sources (radio, television, text messages to phones, and newspapers or magazines):**			
no exposure(ref.)	1.00		
exposed to only one media source	1.023(0.860 to 1.217)	0.090	0.797
exposed to two to three media sources	1.158(0.953 to 1.406)	0.115	0.140
exposed to all the media sources	1.714(1.090 to 2.695)	0.395	0.020**
**Decision making on visitation to family and relatives:**			
husband/partner alone(ref.)	1.00		
women alone	1.162(0.942 to 1.434)	0.124	0.161
joint decision making between women and husband/partner	1.103(0.927 to 1.313)	0.098	0.269
**vVisitation by field workers, and a possible discussion about family planning, in the past 12 months before the survey:**			
no(ref.)	1.00		
yes, but family planning was not discussed	1.239(0.995 to 1.543)	0.139	0.055*
yes, and family planning was discussed	1.291(1.008 to 1.653)	0.163	0.043**
** Employment status of the women in the last 12 months prior to the survey, type of earnings, and the control of cash earnings:**			
no, not employed and those employed, but either not paid at all or paid in kind (ref.)	1.00		
yes, employed and paid in cash, but the control of cash earnings mainly done by the husbands/partners	1.054(0.773 to 1.438)	0.167	0.739
yes, employed and paid in cash, but the control of cash earnings done only by the women.	1.039(0.885 to 1.219)	0.085	0.641
yes, employed and paid in cash, but decision about the cash earnings jointly done by the women and their husbands/partners.	1.208(0.986 to 1.480)	0.125	0.068*
***Community-level (level 2) variables***			
**Place of residence:**			
rural (ref.)	1.00		
urban	1.174(0.994 to 1.386)	0.099	0.059*
POOR(%); 80%IOR	44.8; 0.246 to 5.594		
** Community median age at first marriage/cohabitation:**			
low median age (ref.)	1.00		
high median age	1.236(0.954 to 1.600)	0.163	0.108
POOR(%); 80%IOR	43.3; 0.259 to 5.889		
**Community median ideal number of children:**			
high median number (ref.)			
low median number	0.871 (0.719 to 1.055)	0.085	0.158
POOR(%); 80%IOR	45.6; 0.183 to 4.150		
** Community level of education for women:**			
low educational level (ref.)	1.00		
high educational level	1.608 (1.289 to 2.006)	0.181	<0.001***
POOR(%); 80%IOR	34.8; 0.337 to 7.662		
**Communities within states with CRLs, or not:**			
yes(ref.)	1.00		
no	1.258 (0.622 to 2.546)	0.452	0.522
POOR(%); 80%IOR	42.5; 0.264 to 5.994		
** Community exposure of women to family planning messages through at least one of the different media sources (radio, television, text messages to phones, and newspapers or magazines):**			
low exposure level (ref.)	1.00		
high exposure level	1.255 (1.031 to 1.527)	0.126	0.024**
POOR(%); 80%IOR	42.5; 0.263 to 5.980		
** Community wealth index:**			
high poverty level(ref.)	1.00		
low poverty level	1.067 (0.876 to 1.300)	0.107	0.521
POOR(%); 80%IOR	48.0; 0.224 to 5.084		
**Community level of employment for women:**			
low employment level(ref.)	1.00		
high employment level	1.111(0.891 to 1.385)	0.125	0.349
POOR(%); 80%IOR	46.4; 0.233 to 5.294		
** Geopolitical zones:**			
North Central zone(ref.)	1.00		
North East zone	1.373 (0.685 to 2.752)	0.486	0.371
POOR(%); 80%IOR	39.7; 0.288 to 6.542		
North West zone	1.552 (0.733 to 3.287)	0.594	0.251
POOR(%); 80%IOR	35.9; 0.326 to 7.395		
South East zone	0.360 (0.236 to 0.550)	0.078	<0.001***
POOR(%); 80%IOR	27.8; 0.076 to 1.715		
South West zone	0.756 (0.558 to 1.023)	0.117	0.070*
POOR(%); 80%IOR	40.9; 0.159 to 3.602		
South South zone	0.482 (0.371 to 0.625)	0.064	<0.001***
POOR(%); 80%IOR	27.4; 0.101 to 2.297		
**Constant**	0.008 (0.002 to 0.026)	0.005	<0.001***
***Random effect***			
	**Estimate (95%CI)**	SERs	
**Random intercept variance (RIV)** [RIV for model As^b^ = 1.162(0.973 to 1.387), SERs = 0.105]	0.742 (0.600 to 0.918)	0.080	NA
***General contextual effect***			
**ICC (%) = VPC (%)**	18.4		
**PCV(%)**	36.1		
**MOR**	2.274		
***Postestimation diagnosis***			
**Goodness of fit (Wald test)**			
F statistics	Model Ds vs. model As = 10.29		
p value	<0.001***		

Note: Ref. = the reference subcategory for each variable; aOR = adjusted odds ratios; 95% CI = 95% confidence interval; SERs = standard errors; NA = not available; ICC = intracluster correlation coefficient; VPC = variance partition coefficient; PCV = proportion of change in variance; MOR = median odds ratio; POOR = proportion of opposed odds ratios; 80% IOR = 80% interval odds ratios; vs. = versus/in comparison; and CRLs = cultural and religious laws.

‘*’ = significant at 10% level (p < 0.10);

‘**’ = significant at 5% level (p < 0.05); and

‘***’ = significant at 1% level (p < 0.01).

^a^Final model for the sensitivity analysis, which was carried out using the Demographic and Health Survey list of modern contraceptive methods; and ^b^null model for the sensitivity analysis, which was also carried out using the Demographic and Health Survey list of modern contraceptive methods.

Source: Authors’ computation using Stata version 15.0.

The publisher apologizes for the errors.
