# Supplementary material for: An empirical analysis of the demand for family planning satisfied by modern methods among married or in-union women in Nigeria: Application of multilevel binomial logistic modelling technique
Source: PLoS One. 2024 Mar 21;19(3):e0300744. doi: 10.1371/journal.pone.0300744 (PMC10956820; doi:10.1371/journal.pone.0300744)
Supplement: Attachment — Submitted filename: A rebuttal letter.docx [file pone.0300744.s001.docx]

Emomine Odjesa,

Centre of Excellence in Reproductive Health Innovation (CERHI),

University of Benin, Benin City, Edo State, Nigeria.

19^th^ February 2024

Jianhong Zhou,

Staff Editor,

PLOS ONE.

Dear Sir/ma,

Response to reviewers and editors

# I am writing you this letter in order to respond to the comments made by reviewers and the staff editor regarding my manuscript titled ‘An empirical analysis of the demand for family planning satisfied by modern methods among married or in-union women in Nigeria: Application of multilevel binomial logistic modelling technique’. According to the editor, minor revisions are required.

We have heeded to the comments made by the editors. In relation to this, I have made sure that the reference list is complete and correct. I have also made sure that the papers cited, and hence included in the reference list, are current; that is, no retracted papers were included. In relation to this, the reference number [21] was changed to a relevant current paper. I have also, again, done a thorough general English language editing using a language editing software called ‘curie’. The British English language was the language of choice used for the editing.

We have also heeded to the comments made by the second reviewer. Reviewer two asked for an initial explanation of all the abbreviations/acronyms used. This I have also carefully done. For the specific ones pointed out, ‘MC’ was first used and written in full in line 96, while NFP was first used and written in full in line 101. In addition, I have also included a sub-heading labelled ‘study setting’ as part of the materials and method section to explain the setting and location of the study, as required by reviewer two to be done. Regarding stating the eligibility criteria of the sample, as required by the second reviewer to be done, several additional words were included under the already existing sub-heading ‘the study’s analytical sample’. From line 274 to 280, the following statement was included: *Based on the aims of this study, concentration was done on the married or in-union WRA in Nigeria with a DFP (that is, the totality of NFP), who were also usual residents of the communities in which they were surveyed. This clearly depicts the eligibility criteria for the women to be included in this study and thus represents this study’s analytical sample.* *Note that only the usual residents of the communities surveyed by the 2018 NDHS were concentrated on in this study because it allowed for examining the possible contextual influences on the likelihood of Nigerian married or in-union WRA with a DFP using MC.*

Furthermore, from line 319 to 328, the following statement was included under the already existing sub-heading ‘the study’s analytical sample’: *Additionally, since this study’s analytical sample was obtained from the 2018 NDHS and because of the sample design of this NDHS, as explained previously, this study’s analytical sample thus covers the entire territory of Nigeria. This analytical sample is therefore representative of the Nigerian population structure, together with its naturally occurring hierarchies. This thus allows for, using appropriate statistical analysis, the distinguishing of individual-level and community-level factors, together with the estimation of the different variances at the different levels, that can possibly influence the likelihood of married or in-union women having mDFPS. Also, it allows for, using appropriate statistical techniques, the generalisation of our result findings from this sample into the Nigerian population. Thus, this analytical sample can be used to meet the aims of this study.*

In addition, the second reviewer mentioned that the limitation of the non-generalisation of the results, globally, should also be included, because the study was limited to only Nigeria. This was included in line 889 to 895 under the already existing sub-heading ‘limitation of the study’ as part of the discussion section: *Additionally, this study is limited to Nigeria, and the outcome thus cannot be generalised globally. However, because the DHS has standardised methodology and data collection processes across countries and has consistent content over time, which in all also leads to the presence of similar variables in the DHS data across and within countries over time, this study can be easily replicated in other African or developing countries where DHS data are available[69]. This approach allows for comparisons of the results across countries and hence across different populations, both cross-sectionally and over time, and even within a country over time[69].*

I have also carefully noted under the discussion, in fact in the entire length of this paper, that the study is based on married or in-union women, not just women, of reproductive ages in Nigeria, as mentioned to be done by the second reviewer. I also carefully checked the introduction section, in fact the entire length of this paper, to make sure I was not repeating words, sentences, or ideas, as mentioned to be done by the second reviewer.

Also, as previously noted in the first rebuttal letter I sent, I changed the data availability statement on the application portal online to ‘some restrictions apply to data availability’. This was done because accessing the data requires making a formal written request at the DHS website (https://dhsprogram.com/data/dataset/Nigeria_Standard-DHS_2018.cfm?flag=0). Upon getting a written approval, the data set can then be freely downloaded. In fact, from the following link, you can clearly see the conditions required to use the DHS dataset: https://dhsprogram.com/data/terms-of-use.cfm

Thank you very much for taking time out to review my initial application, the first revision, and also this present revision.

Yours faithfully,

Emomine Odjesa.
